# Undergraduate orthopedic education: Is it adequate?

**DOI:** 10.4103/0019-5413.45328

**Published:** 2009

**Authors:** Jagdish Menon, Dilip K Patro

**Affiliations:** Department of Orthopaedics, Jawaharlal Institute of Medical Education and Research (JIPMER), Pondicherry - 605 006, India

**Keywords:** Curriculum, undergraduate, medical education

## Abstract

**Background:**

Basic musculoskeletal knowledge is essential to the practice of medicine. The purpose of this study was to assess the adequacy of musculoskeletal knowledge of medical students.

**Materials and Methods:**

The validated basic competency examination in musculoskeletal medicine devised by Freidman and Bernstein was administered to final year medical students just prior to their final professional examination. Participants were also required to assess their confidence at making a musculoskeletal physical examination and diagnosis as well as comment on the adequacy of time in the curriculum devoted to Orthopedics.

**Results:**

The response rate was 83% (40/48). The average cognitive examination score was 48.3%. Two participants (5%) obtained a score of ≥ 73.1%, the recommended mean passing score. Seventeen students (42.5%) felt orthopedic clinical cases were the most difficult to perform a physical examination and diagnose. Thirteen students (32.5%) felt that the time devoted to orthopedics in the medical curriculum was inadequate.

**Conclusions:**

Ninety-five percent of the students failed to show basic musculoskeletal competency. A change in medical curriculum and teaching methods is required to address this problem.

## INTRODUCTION

Disorders of the musculoskeletal system are the commonest reason why a person seeks medical aid.^1^ Recognizing the enormous burden of musculoskeletal diseases, the World Health Organization designated the first decade of the twenty first century as The Bone and Joint Decade.^2^ Primary care doctors, physicians, pediatricians, rheumatologists, physiatrists, emergency care providers and orthopedic surgeons form part of musculoskeletal care services. Fundamental knowledge of musculoskeletal disorders is essential to the practice of medicine. Graduate medical education should provide a strong foundation for diagnosing and treating musculoskeletal disorders. Recent studies have shown that decrease in physician competency in musculoskeletal medicine is probably due to educational deficiencies at the medical school level.^3^–^11^

Freedman and Bernstein (1998), developed a validated musculoskeletal examination to test health care providers in the basics of musculoskeletal disorders. They found that 82% of first-year postgraduate residents failed to demonstrate adequate basic cognitive understanding of musculoskeletal problems.^3^ Similar findings have been reported from other parts of the world.^5^,^8^ The purpose of this study is to determine the adequacy of orthopedic learning at graduate level by testing final year undergraduate students, just prior to their final professional examination.

## MATERIALS AND METHODS

The validated basic cognitive test consisting of 25 short answer questions was administered to the final MBBS (IX semester students) just prior to their final professional examination. The test was timed in such a way that all students had completed their clinical postings. No prior knowledge of this examination was provided to the students. No student had attended a similar examination in the past. All participants took the examination voluntarily. Students absent for that particular class and those who voluntarily choose not to take the examination were excluded. The examination was conducted in the same fashion to all the participants. No time limit was enforced for completion of the examination. The answer sheets were collected promptly and scored anonymously by the same faculty. Each question was worth one mark. Partial credit was given to questions with multiple answers. Raw scores were multiplied by 4 to give percentage scores. Overall scores and responses to individual questions were analyzed. The recommended passing score as suggested by Freedman and Bernstein of ≥ 73.1 was adhered to. Students were also asked to rate their ability to make a physical diagnosis and diagnosis in final professional clinical subjects (i.e., Medicine, Surgery, Obstetrics and Gynecology, Pediatrics and Orthopedics) on a scale of 1 to 5 in increasing order of difficulty. Additionally, students were required to comment on whether the time devoted to orthopedics in the curriculum was adequate [Appendix A].

## RESULTS

Of the 48 students present, 8 voluntarily choose not to participate. Forty students underwent the examination. The average cognitive score was 48.3%. Only two (5%) of the participants obtained a score of ≥ 73.1%. The student mean score for each question is shown in Table 1. These ranged from 95% for the question on carpal tunnel syndrome (Question No. 10) to 7.5% for metabolic bone diseases (Question No. 19). The questions were also separated in to general orthopedics, trauma and anatomy groups. The average score in the general orthopedics, trauma and anatomy groups were 72.0%, 74.5% and 57.2%, respectively. Seventeen students (42.5%) felt orthopedic clinical cases were the most difficult to perform a physical examination and diagnose. None of the students ranked orthopedics as number 1, i.e., easiest clinical specialty to perform a physical examination and diagnose. Twenty-seven (62.5%) students felt that the time allocated to orthopedics in the medical curriculum was sufficient.

**Table 1:** Mean student scores

Question	Answer	Mean score
1. What common problem must all newborns be examined for?	Congenital dislocation of hip (CDH, dislocation; subluxation also accepted): 1 point	80.0
2. What is compartment syndrome?	Increased pressure in a closed fascial space: 1 point	82.5
3. Acute septic arthritis of the knee may be differentiated from inflammatory arthritis by which laboratory test?	Any analysis of fluid from aspiration (cell count, gram stain, culture): 1 point	37.5
4. A patient's dislocates his knee in a car accident. What structure/s is/are at risk for injury and therefore must be evaluated?	Must mention popliteal artery: 1 point	47.5
5. A patient punches his companion in the face and sustains a fracture of the 5th metacarpal and a 3-mm break in skin over the fracture. What is the correct treatment and why?	Irrigation and debridement; risk of infection: ½ point each	17.5
6. A patient comes to the office complaining of low-back pain that wakes him from sleep. What two diagnoses are you concerned about?	Tumor and infection: ½ point each	38.75
7. How is compartment syndrome treated?	Fasciotomy (surgery also accepted): 1 point	92.5
8. A patient lands on his hand and is tender to palpation in the snuff box (the space between the thumb extensor and the adductor tendons). Initial radiographs do not show a fracture. What diagnosis must be considered?	Scaphoid fracture (carpal bone fracture also accepted): 1 point	75.0
9. A 25-year-old male is involved in a motor vehicle accident. His left limb is in a position of flexion at the hip and knee with internal rotation and adduction at the hip. What is the most likely diagnosis?	Hip dislocation: 1 point	60.0
10. What nerve is compressed in carpal tunnel syndrome?	Median nerve: 1 point	95.0
11. A patient has a disc herniation pressing the 5th lumbar nerve root. How is the motor function of the fifth lumbar root tested?	Dorsiflexion of the great toe (toe extensors also accepted): 1 point	20.0
12. How is the motor function of the median nerve tested in the hand?	Any median nerve function (Metacarpophalangeal finger flexion; thumb opposition, flexion or abduction): 1 point	35.0
13. A 12-year-old boy severely twists his ankle. Radiographs show only a soft tissue swelling. He is tender at the distal aspect of the fibula. What are the two possible diagnoses?	Ligament sprain and Salter Harris 1 fracture (sprain, fracture also accepted): ½ point each	36.25
14. A patient presents with new onset low back pain. Under what conditions are plain radiographs indicated? Please name five (example: history of trauma)	Age > 50 years; neurological deficit; bowel or bladder changes; history of cancer; drug use or steroid use; systemic symptoms (night pain, fever); pediatric population: ½ point each	50.0
15. A patient has a displaced fracture near the fibular neck. What structure is at risk for injury?	Common peroneal nerve (peroneal nerve also accepted):1 point	62.5
16. A 20-year-old injures his knee while playing football. You see him in the same day and he has a knee effusion. An aspiration shows frank blood. What are the three common diagnoses?	Ligament tear, fracture, peripheral meniscal tear (capsular tear, patellar dislocation also accepted): ½ point each, full credit for 2 correct responses	23.75
17. What are the five common sources of cancer metastasis to the bone?	Breast, prostate, lung, kidney, thyroid: ¼ point each, full credit for four correct responses	68.75
18. Name two differences between rheumatoid arthritis and osteoarthritis?	Any two correct statements (e.g., inflammatory vs. degenerative, proximal vs. distal interphalangeal joint, etc.): ½ point each	67.5
19. What malignancy may be present in bone yet typically is not detected on the bone scan?	Myeloma (full credit for hematological malignancies- leukemia, lymphoma): 1 point	12.5
20. What is the function of the normal anterior cruciate ligament of the knee?	To prevent anterior translation of the tibia on the femur: 1 point	17.5
21. What is the difference between osteoporosis and osteomalacia?	Osteoporosis – decreased bone density; osteomalacia – decreased bone mineralization (any true statement about epidemiology, pathophysiology, e.g., estrogen vs. vitamin D also accepted): 1 point	7.5
22. In elderly patients, displaced fractures of the neck of femur are typically treated by joint replacement, whereas fractures of the trochanter are treated by plate and screws. Why?	Blood supply to femoral head (avascular necrosis, nonunion also accepted): 1 point	52.5
23. What muscle/s is/are involved in lateral epicondylitis?	Wrist extensors (full credit for any wrist extensor – extensor carpi radialis brevis, extensor carpi radialis longus, extensor digitorum communis): 1 point	40.0
24. Rupture of the biceps at the elbow results in weakness of both elbow flexion and ……………?	Supination: 1 point	42.5
25. What muscle(s) control(s) external rotation of the humerus with the arm by the side?	Infraspinatus or teres minor accepted(full credit): 1 point	37.5

## DISCUSSION

Fundamental musculoskeletal knowledge is essential to clinical practice. Primary care physicians have been found to be deficient in orthopedic knowledge and skills.^12^,^13^ In a survey of family physicians, it was found that half of them thought that their training in orthopedics was inadequate.^14^ Physicians who underwent prolonged training in orthopedics had substantially higher confidence in their ability to diagnose and treat musculoskeletal conditions.^15^–^17^

While the widespread impact of musculoskeletal disease on society is indisputable, the relative inattention this subject has received in undergraduate education has been acknowledged.^18^ A comprehensive review of the curricula indicated a marked discrepancy between the necessary skills and knowledge to treat patients with musculoskeletal disorders and the amount of time devoted to teaching these skills in Canadian medical schools. While 27.4% of primary care practice involved musculoskeletal disorders, only 2.26% of curriculum in a typical Canadian medical school was devoted to mandatory musculoskeletal education.^4^ Almost half of American medical schools do not require formal clinical or basic musculoskeletal course prior to graduation.^7^ In India, orthopedic teaching forms 3.7% of the total undergraduate medical curriculum.^19^

In an attempt to verify the adequacy of musculoskeletal training, Freidman and Bernstein developed a basic competency examination. Twenty-five short answer questions were framed keeping in mind the commonly occurring problems encountered in primary care. These included fractures and dislocations, low back pain and osteoarthritis. The examination also covered emergencies that required immediate referral to an orthopedic surgeon as well as basic anatomical knowledge necessary for physical diagnosis. The questions were validated by chair persons of both orthopedic and internal medicine residency programmes who recommended the mean passing grade of ≥ 73.1.^6^ The test was then administered to first-year postgraduates of different specialties. The mean score achieved was 59.6 ± 12%. Seventy residents (82%) failed to demonstrate basic competency in the examination.^3^ This examination was developed as a competency examination but was later relabeled as a “cognitive examination” as it tests more of quality of knowledge than skills.^8^

In a similar examination administered to 22 medical students in their last month of training at Barbados, West Indies, 82 percent of the students scored below the recommended passing score. The questions were also categorized into anatomy, trauma and general orthopedics sections. None of the students passed the anatomy section of the examination, whereas 64% failed in trauma and 45% in general orthopedics.^5^

A pass rate of 39% was reported when the same examination was administered to interns in Australia with a mean score of 69.4 ± 12.0%. The score improved to 77 ± 10.9% when general practioners were examined (passing rate of 68%).^8^

Medical students, residents and staff physicians from Hawaii were tested with the same cognitive examination. Seventy-nine percent of the participants failed the competency examination. Scores were significantly better in those participants who had additional exposure to orthopedics at medical school level.^10^ The Harvard medical school tested medical students using the Freidman Bernstein examination and a separate 30 question survey. The pass rate was 7%. Additionally 54% of the students felt low to adequate level of confidence in performing a musculoskeletal physical examination.^11^

The low scores in our study probably reflect that the test was administered earlier, i.e., in the final year. Since basic medical education is not complete without internship, we feel better scores could be achieved if a similar test was administered on interns. Nevertheless the inadequacy of musculoskeletal education stands exposed. The low scores could also be an indicator of the importance given to orthopedics, as a subject in the medical curriculum. Our study also demonstrates poor scores in anatomy questions with the average score being 57.2%. With the change in under graduate medical education rules by the Medical Council of India in 1997, Anatomy in spite of being a core subject find its time reduced by 30%.^19^ Time devoted to anatomic dissection and other traditional methods of instruction is decreasing.^20^,^21^ We feel a rethink is required regarding the time and nature of instruction in Anatomy. Seventeen of our students (42.5%) felt that orthopedics was the most difficult specialty to make a clinical diagnosis. The low level of confidence felt by students while performing orthopedic physical examination and diagnosis was studied.^11^ Using the same scale to compare confidence while performing musculoskeletal physical examination and a respiratory system examination; it was found that students who were very confident in examining the respiratory system felt low to average confidence while examining the musculoskeletal system. Eighty-six percent of these students recommended that more time be devoted to their current musculoskeletal curriculum. In our study, however, 62.5% of students felt that time devoted to orthopedics was sufficient. We feel that our students were assessing subjects from an examination point of view, rather than importance in clinical practice. One student wrote that “the teaching is sufficient for the 60 marks of theory and one short case which is the required in the Orthopedics MBBS examination”. Another student mentioned “Orthopedics is low priority due to the higher marks awarded to other final MBBS Subjects”. Orthopedics therefore must be accorded the status of a separate subject in the medical curriculum with independent evaluation similar to ENT or Ophthalmology. Merely expanding the curriculum is not sufficient; the examination process must be more relevant.

Results from our study should be viewed within the context of various limitations. The Freidman Bernstein questionnaire was not validated for relevance in our country. We noticed that musculoskeletal infections were not included in the questionnaire. We personally felt that infection and congenital anomalies deserved better representation. The examination, however, was not modified so as to have a better comparison with other studies. The results show the trends from one medical institution. We suggest that a similar study is needed with questionnaire arranged in consideration of the clinical workload available in our country. A larger student population would be necessary for more significant results. The examination may not have been totally flawless but the overall scores underline the fact that medical students are not adequately prepared in orthopedics.

An increasing burden of musculoskeletal diseases demands that future doctors should be well trained and competent in this field. It is the responsibility of medical institutions to strengthen undergraduate orthopedic education to rectify the current deficiency. These changes will eventually translate into better patient care.

## APPENDIX A

**1. In which clinical specialty do you find it most comfortable to make a physical examination and diagnosis?**

**Table:**

Subject	Rank
Medicine	
Surgery	
Obstetrics and gynaecology	
Pediatrics	
Orthopedics	

Rank from 1 to 5 in ascending order of difficulty with 1 as easiest and 5 as most difficult specialty to make a physical examination and diagnosis.

**2. Do you feel that the time devoted to Orthopedics in medical curriculum is sufficient?**

**Table:**

Good (More than enough classes and clinics)	□
Adequate (Enough classes and clinics, more welcome)	□
Inadequate (More classes and clinics definitely required)	□
Poor (Time grossly inadequate)	□

Tick One response
